# Combined Patellofemoral Arthroplasty With Patellar Realignment: Surgical Technique and Clinical Outcomes in a Retrospective Case Series

**DOI:** 10.1016/j.artd.2025.101951

**Published:** 2026-01-24

**Authors:** Paul B. Walker, Lisa Su, Mathangi Sridharan, Murray Wong, Matthew Dipane, Guillermo Araujo-Espinoza, Kristofer J. Jones, Adam A. Sassoon

**Affiliations:** Department of Orthopaedic Surgery, University of California, Los Angeles, CA, USA

**Keywords:** Patellofemoral arthroplasty, MPFL reconstruction, Tibial tubercle osteotomy, Patellofemoral arthritis, Patellar instability

## Abstract

**Background:**

Isolated patellofemoral joint arthritis with patellar malalignment in young patients presents a unique challenge, as these patients typically wish to avoid early total knee arthroplasty. The purpose of this retrospective case series is to describe a standardized dual-surgeon technique for combined patellofemoral arthroplasty (PFA) with patellar realignment using medial patellofemoral ligament reconstruction or tibial tubercle osteotomy and to evaluate early clinical and patient-reported outcomes.

**Methods:**

A retrospective review was conducted on patients who underwent combined PFA and patellar realignment by a fellowship-trained arthroplasty surgeon and fellowship-trained sports surgeon. Outcomes including implant survivorship, functional outcomes, complications, and patient-reported measures (Knee Injury and Osteoarthritis Outcome Score Joint Replacement, Patient Reported Outcomes Measurement Information System (PROMIS), Global Physical Health, PROMIS Global Mental Health, and Forgotten Joint Score) were assessed.

**Results:**

Eleven knees in 9 patients were included (55.5% female; median age 41 ± 13.4 years; median body mass index 26 ± 6.2). All knees had isolated patellofemoral arthritis. Nine knees underwent PFA with medial patellofemoral ligament reconstruction for instability or dislocation, while 2 underwent PFA with tibial tubercle osteotomy for patella alta. Two patellae were chronically dislocated, additionally requiring lateral release. Six knees had prior failed stabilizing procedures. The mean operative time was 121 minutes (94–161), with a mean follow-up of 24.0 ± 11.5 months. One patient experienced a periprosthetic patellar fracture at 10 months, followed by a refracture at 14 months, requiring open reduction and internal fixation and extensor mechanism repair. No cases of re-dislocation, maltracking, infections, wound complications, or other medical issues occurred. Knee Injury and Osteoarthritis Outcome Score Joint Replacement scores improved by an average of 14.8 ± 11 points.

**Conclusions:**

Combined PFA and patellar realignment surgery can be done efficiently and is associated with improved patient-reported outcomes, with complications limited to a single case of periprosthetic patellar fracture.

## Introduction

Isolated patellofemoral arthritis, estimated to affect approximately 10% of individuals with radiographic knee arthritis, presents a significant challenge for orthopaedic surgeons. This condition is particularly complex in younger, more active patients and requires distinct management considerations compared to tricompartmental osteoarthritis [1,2]. For patients with patellar maltracking or malalignment, repetitive trauma is likely to lead to isolated patellofemoral arthritis, with minimal progression in other knee compartments [3]. As a result, these patients are often candidates for surgical intervention focused solely on the patellofemoral joint.

Patellofemoral arthroplasty (PFA) was first introduced in the 1950s, but early prostheses had significant limitations. Suboptimal trochlear implant geometry led to problems with patellar tracking, instability, and impingement, contributing to early failure [4]. With more recent designs, implant survivorship and outcomes have improved. Today, the most common cause of failure is the progression of osteoarthritis; however, patellar maltracking still contributes to approximately 10% of all failures, which remains significant [2,5].

Patellar realignment procedures, such as medial patellofemoral ligament (MPFL) reconstruction and tibial tubercle osteotomy (TTO), are commonly indicated for patients with recurrent patellar instability to improve patellar tracking and prevent future osteoarthritis [6]. Previous descriptions of PFA with MPFL reconstruction or TTO have been described in small case series, with promising short-term results [[7], [8], [9]]. This study presents short-term outcomes of patients at our institution who underwent combined PFA and patellar realignment surgery. MPFL reconstruction was performed for recurrent patellar dislocations or instability, while TTO was performed in cases of patella alta. In contrast to previous studies, the PFA component was performed by a fellowship-trained arthroplasty surgeon, while a fellowship-trained sports surgeon conducted the patellar realignment. We hypothesize that concomitant PFA and patellar realignment surgery could improve functional outcomes and implant survivorship in patients with isolated patellofemoral arthritis and patellar maltracking.

## Materials and methods

After obtaining institutional review board approval (IRB #23-001918), a retrospective review was conducted of a consecutive series of patients who underwent combined PFA and patellar realignment procedures between July 2021 and May 2023. Informed consent was obtained from all included patients. The inclusion criteria included patients who underwent the combined procedure with a minimum follow-up period of 24 months. Exclusion criteria included advanced tibiofemoral osteoarthritis (Kellgren–Lawrence [KL] grade > II), inflammatory arthropathy, fixed flexion contracture >10°, coronal malalignment >5° varus or valgus, body mass index (BMI) >40 kg/m^2^, ligamentous instability, and active infection.

Patient demographics, including age, gender, BMI, and American Society of Anesthesiologists status, were collected. Outcome measures were categorized as perioperative, short-term postoperative, and short-term survivorship outcomes. Perioperative outcomes included operative time, tourniquet time, estimated blood loss, length of hospital stay, and discharge disposition. Short-term postoperative outcomes, assessed through 90 days, included wound complications, periprosthetic fracture, infection, arthrofibrosis, recurrent maltracking or instability, venous thromboembolism, range of motion, and other medical complications. Short-term survivorship outcomes included implant survivorship, reoperation and revision rates, recurrent instability, and patient-reported outcome measures (PROMs) assessed at a minimum 2-year follow-up.

PROMs assessed in this study included the Knee Injury and Osteoarthritis Outcome Score Joint Replacement, Patient Reported Outcomes Measurement Information System (PROMIS) Global Physical Health (GPH), PROMIS Global Mental Health, and the Forgotten Joint Score.

**Figure 1:**
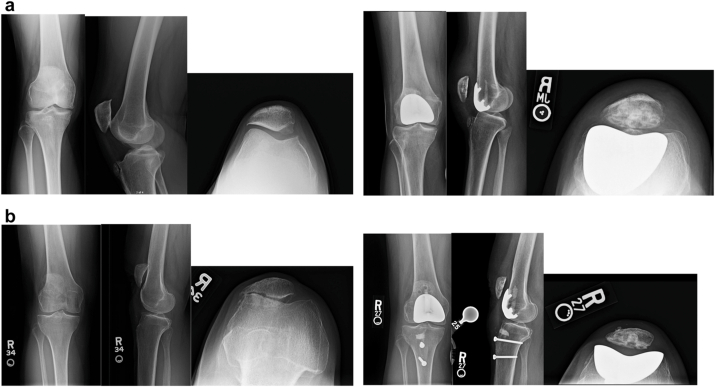
Representative preoperative and postoperative radiographs: (a) patellofemoral arthroplasty (PFA) with medial patellofemoral ligament (MPFL) reconstruction, (b) patellofemoral arthroplasty (PFA) with tibial tubercle osteotomy (TTO).

All radiographic and magnetic resonance imaging (MRI) measurements were obtained from preoperative imaging. Radiographic assessments included the Caton–Deschamps Index (CDI) for patellar height, KL grading for tibiofemoral and patellofemoral osteoarthritis severity, and patellar tilt measured on standardized weight-bearing anteroposterior, lateral, and Merchant radiographs. MRI was used to evaluate the congruence angle, Sperner patellofemoral joint classification, Dejour classification of trochlear dysplasia, and tibial tubercle–trochlear groove (TT–TG) distance. Patellar maltracking was defined according to commonly accepted thresholds, including patellar tilt > 5° on Merchant view, a positive congruence angle (>16° considered abnormal), or TT–TG distance > 20 mm. All measurements were independently performed by 2 fellowship-trained orthopaedic surgeons (P.W. and L.S.), with discrepancies adjudicated by a third reviewer (M.S.). Interclass correlation coefficients were calculated for all radiographic parameters and demonstrated high interobserver reliability (interclass correlation coefficient >0.85 for all measurements). Table 1 summarizes the preoperative imaging characteristics and surgical details for patients undergoing PFA with MPFL reconstruction and PFA with TTO. Representative imaging is shown in Figure 1.

**Table 1:** Individual patient data including surgical procedure, preoperative radiographic measurements, alignment, comorbidities, and prior surgical history.

Patient ID	Surgical procedure	Preop CDI (Caton–Deschamps) Index	Preop congruence angle	Kellgren-Lawrence PF	Sperner PFJ	Dejour	TT-TG (mm)	Preop patellar tilt (deg)	Postop patellar tilt (deg)	Alignment	Comorbidities	Prior surgery
1	PFA and MPFL reconstruction	1.3	25	2	3	C	18	3.3	5	Neutral	Bipolar, depression	MPFL
2		1.2	8	3	3	A	9	−0.7	−1	Neutral	PTSD, HLD, OSA	NA
3		0.9	20	3	3	C	9.5	24.6	0.5	Neutral	Asthma	MPFL
4		0.5	100	4	4	D	13	54.1	5.7	Mild valgus	Depression	Fulkerson osteotomy
5		0.6	102	4	4	D	13	59.5	−4.3	Mild valgus	Depression	Fulkerson osteotomy
6		1.1	16	2	2	C	12	9.5	8.2	Neutral	Hypertension	NA
7		1.2	21	4	4	C	20	15.3	4	Mild varus	NA	MPFL
8		1.2	13	2	2	B	14	7.7	4.3	Neutral	IBS	NA
9		1.3	−17	4	4	B	27	7.1	3.2	Moderate valgus	Cerebral palsy (GMFCS II)	MPFL + TTO
10	MPFL and TTO	1.6	18	4	3	B	20	13.2	−3	Neutral	HLD	NA
11		1.6	6	4	3	NA	NA	9.4	−2.3	Neutral	HLD	NA

Alignment categories: neutral (178°–182°), varus (<178°: mild 174–177°, moderate 170–173°, severe <170°), valgus (>182°: mild 183–186°, moderate 187–190°, severe >190°).

PF, patellofemoral; PFJ, patellofemoral joint; HLD, hyperlipidemia; OSA, obstructive sleep apnea; IBS, irritable bowel syndrome; GMFCS, Gross Motor Function Classification System; NA, not available; PTSD, post-traumatic stress disorder.

Preoperative metrics include Caton–Deschamps Index (CDI), congruence angle, Kellgren-Lawrence (KL) grade, Sperner classification of patellofemoral (PF) osteoarthritis, Dejour classification of trochlear dysplasia, tibial tubercle-to-trochlear groove (TT-TG) distance, and patellar tilt (preoperative and postoperative). Alignment is defined by the hip-knee-ankle (HKA) angle.

### Statistical analysis

Descriptive statistical analysis was performed using Microsoft Excel (Redmond, WA). Continuous data were reported as means with standard deviations, while categorical data were presented as counts and percentages.

### Patient selection

All patients underwent a standardized clinical evaluation, including assessment of patellar tracking, apprehension, and passive lateral translation in full extension and at 30° of knee flexion. All patients demonstrated positive findings on examination. Patellar instability was defined as a documented history of recurrent lateral patellar dislocations or symptomatic subluxations despite nonoperative management or prior failed stabilization procedures. Each patient had experienced at least 2 discrete, documented lateral patellar dislocations in the same knee.

Radiographic and MRI evaluation confirmed isolated patellofemoral arthritis with preservation of the medial and lateral tibiofemoral compartments. Patella alta was defined radiographically by a CDI greater than 1.2. The primary indication for PFA was severe anterior knee pain and functional limitations attributable to patellofemoral joint degeneration seen on imaging. MPFL reconstruction and/or TTO were performed in the setting of structural malalignment or instability necessitating realignment. Intraoperatively, all patients demonstrated full-thickness cartilage loss of the patella and trochlea, with intact weight-bearing cartilage in both the medial and lateral tibiofemoral compartments.

Patients with advanced tibiofemoral osteoarthritis were excluded. Specifically, a KL grade greater than II in the tibiofemoral compartment was considered a contraindication to isolated PFA. KL grade II changes were acceptable for PFA if symptoms and exam were clearly attributable to the patellofemoral joint, even in the absence of radiographically severe disease. Patients were also deemed unsuitable if they had inflammatory arthropathy, fixed flexion contracture greater than 10°, coronal malalignment exceeding 5° varus or valgus, BMI greater than 40, or ligamentous insufficiency.

Overall, patients were generally healthy, with comorbidities detailed in Table 1. Four patients presented with bilateral patellofemoral disease. Two of these patients underwent planned staged bilateral procedures within the study period, and both knees met the minimum follow-up requirement and were therefore included in the analysis. The remaining 2 patients subsequently underwent contralateral surgery after completion of the study window; however, these contralateral knees were not included due to insufficient postoperative follow-up at the time of data collection. At most recent clinical follow-up, both latter patients report that their nonincluded contralateral knees are doing well clinically with high satisfaction.

### Surgical technique

Patients were positioned supine with a well-padded proximal thigh tourniquet. Examination under anesthesia documented range of motion, tracking, and degree of instability. The PFA portion of the procedure was performed by a fellowship-trained arthroplasty surgeon, while the patellar stabilization portion was performed by a fellowship-trained sports medicine surgeon.

A medial parapatellar arthrotomy was used in all cases, and great care was taken to avoid damage to the underlying condylar cartilage and anterior horn of the medial meniscus at the distal extent of the arthrotomy. The joint was inspected to confirm full-thickness cartilage loss within the patellofemoral compartment with preservation of tibiofemoral compartments. The anterior trochlear resection was performed utilizing an intramedullary-based cutting guide. Care was taken to ensure adequate external rotation of the trochlear component. Whiteside’s line and the intercondylar axis were used as reference points, and typically the anterior cut was performed such that 2-3 degrees of additional external rotation was targeted relative to the intercondylar axis to improve patellar tracking. Following the anterior resection, the onlay trochlear component (Zimmer Gender Solutions system, Warsaw, IN) was sized to avoid overhang, while maximizing coverage of the anterior femur. The appropriately sized milling guide for the trochlear component was used to complete the trochlear preparation. Lug preparation for the trochlear component was then performed and the trial implant was impacted in place. Measured patellar resection was then performed to restore patellar thickness while avoiding thinning the patella to less than 13 mm. For combined MPFL reconstruction cases, the anticipated position of suture anchors in the patella was marked so that patellar lug preparation could avoid the anchor pathways. A patellar trial was then placed and patellar tracking was assessed.

In instances of chronic patellar dislocation, lateral releases were performed to allow for reduction of the patella over the trochlea in a position of full extension. Through the same exposure, the lateral gutter was developed and a graded release of the lateral retinaculum in layer 2 was carried out from the superolateral patellar border distally toward the patellar tendon, preserving the capsule when possible; release was titrated until the patella centered without excessive medial translation. Implants were then cemented in place using Rally Cement with gentamicin (Smith and Nephew, Memphis, TN) under tourniquet control with meticulous cement removal. The tourniquet was then deflated before final assessment of patellar tracking to eliminate any quadriceps distortion related to tourniquet tension. Following cement curing, the patellar realignment portion of the procedure was initiated, at which point operative responsibility transitioned to the sports medicine surgeon.

For MPFL reconstruction, the superomedial patellar border was prepared at the native footprint. A tibialis anterior allograft (doubled) was secured to the patella with a suture anchor while avoiding interference with patellar component pegs. The graft was routed between layers 2 and 3 to a femoral tunnel created at Schöttle’s point under fluoroscopic guidance and fixed with an interference screw at approximately 30° of flexion, tensioned to allow one quadrant of lateral translation in extension. This standardized MPFL reconstruction protocol was used in all 9 MPFL cases.

For TTO in patella alta, the midline incision was extended distally. Capsulotomies were made along the patellar tendon borders, and the lateral cortex of the tibia was exposed. An oblique osteotomy with preservation of the lateral cortical hinge was performed. The tubercle segment was then elevated and distalized to normalize patellar height. The osteotomy was provisionally fixed with a K-wire, proximal voids were filled as needed, and final fixation was achieved with 2 4.5 mm cortical screws using lag technique. Bone grafting with MD3T Osteorepair bone void filler was then performed proximal to the distalized segment. Fluoroscopy was used to confirm screw position, absence of fracture, and corrected patellar height.

Knees were cycled repeatedly to verify central tracking without tilt through a full arc of motion following completion of the stabilization procedure. Wounds were irrigated and closed in layers; no drains were used. Venous thromboembolism prophylaxis consisted of aspirin 81 mg twice daily. A hinged knee brace locked in extension was applied for ambulation, with early active and active-assist range of motion per protocol.

Based on a retrospective review of isolated PFA cases performed by the same arthroplasty surgeon over the preceding 5 years, the addition of the stabilization procedure (MPFL or TTO) increased mean operative time by approximately 50 minutes.

### Postoperative management and rehabilitation

Patients were followed at 2 weeks, 6 weeks, 3 months, 6 months, 1 year, and annually thereafter. A hinged knee brace was applied postoperatively. Formal outpatient physical therapy was initiated at 1 week postoperatively, with patients instructed to attend therapy sessions twice weekly. For the first 2 weeks, range of motion was restricted to 0-90° and weight-bearing was permitted with the brace locked in extension. Patients were instructed to use crutches or a walker, with gradual transition to partial and then full weight-bearing as tolerated once quadriceps control was demonstrated.

Between weeks 2 and 6, brace wear continued during ambulation, typically with a lateral buttress in MPFL cases or a locked extension brace in TTO cases. Range of motion was progressed under the supervision of a licensed physical therapist using a standardized rehabilitation protocol, with emphasis on achieving 90° by 6 weeks, avoiding valgus stress, and limiting passive stretching. Patients performed active and active-assist range of motion exercises only, along with quadriceps, hamstring, gluteal, and hip isometric strengthening.

From weeks 6 to 12, patients were generally permitted full weight-bearing with progressive discontinuation of the brace once adequate quadriceps control and normalized gait mechanics were achieved. Range of motion was advanced toward full, with initiation of stationary cycling and closed-chain strengthening, including step-ups and leg press within a safe arc. Proprioceptive and balance training were introduced.

Between 3 and 4 months, patients progressed to functional training, incorporating elliptical and nonimpact conditioning, core and hip strengthening, and early sport-specific drills. From 4 to 6 months, progression included plyometrics and running programs as bone and soft tissue healing permitted. Return to full sport and impact activity was typically considered between 6 and 9 months, guided by functional testing, absence of effusion, symmetric strength (≥85% limb symmetry), and pain-free activity.

## Results

### Patient demographics, surgical metrics, and imaging characteristics

A total of 11 knees in 9 patients were included in the study. The mean age of the patients was 41 ± 13.4 years, with a mean BMI of 26 ± 6.2. Of the patients, 5 (55.5%) were female. The American Society of Anesthesiologists score was 2 ± 0.67. The laterality of the procedures was predominantly left-sided, with 6 (54.5%) of the knees being treated on the left side.

The most common indication for surgery was patellofemoral arthritis with a history of multiple prior patellar dislocations, affecting 9 (81.8%) knees, followed by chronic anterior patellar subluxation or dislocation in 2 (22.2%) knees. A history of prior patellar stabilization procedures was present in 6 (54.5%) of the knees. The mean follow-up time for all patients was 24.0 ± 11.5 months. Demographic data are presented in Table 2.

**Table 2:** Overview of patient demographics and clinical characteristics.

Clinical variable	Mean ± SD	N (%)
Surgical procedures		11
Total patients		9
Age (y)	41 (13.4)	
Female sex		5 (55.5%)
BMI	26 (6.2)	
ASA	2 (0.67)	
Laterality (Left)		6 (54.5%)
Indication		
Multiple prior patellar dislocations		9 (81.8%)
Chronic patellar subluxation or dislocation		2 (22.2%)
Prior patellar stabilization procedure		6 (54.5%)
Mean follow-up time (mo)	24.0 ± 11.5 mo	

ASA, American Society of Anesthesiologists; Left, left extremity.

The mean surgical time from incision to closure was 121.9 ± 21.9 minutes, with a mean tourniquet time of 56.7 ± 23.2 minutes. Estimated blood loss was 154.5 ± 52.2 cc. All surgeries (100%) were performed using a medial parapatellar approach, and a lateral release was performed in 2 cases (18.2%). All patients undergoing PFA + MPFL reconstruction were discharged on the same day of surgery, while the 2 undergoing PFA + TTO were discharged on postoperative day one. Trochlear components ranged from size 2 to 5, with size 4 being most common. Patellar components ranged from 32 mm to 38 mm in diameter, with thicknesses between 8 mm and 9.5 mm. Surgical metrics are displayed in Table 3.

**Table 3:** Surgical metrics, including operative time, tourniquet duration, estimated blood loss, and surgical approach.

Operative variable	Mean ± SD	N (%)
Surgical time incision to closure (min)	121.9 (21.9)	
Tourniquet time (min)	56.7 (23.2)	
EBL (cc)	154.5 (52.2)	
Medial parapatellar approach		11 (100%)
Lateral release		2 (18.2%)

estimated blood loss.

The median preop CDI was 1.2 ± 0.42, suggesting mild patella alta, while the congruence angle had a median of 18.0° ± 37.63, indicating lateral patellar displacement. Most patients had severe osteoarthritis, with a KL median of 4.0 ± 0.90. The median TT–TG distance was 13.5 mm ± 5.62. Trochlear morphology varied among patients: one knee had no dysplasia, while the remaining knees demonstrated varying degrees of trochlear dysplasia, with 3 classified as Dejour type B, one as type A (mild dysplasia), four as type C, and two as type D, indicating a broad spectrum of femoral dysplasia severity across the cohort.

Surgical outcomes showed significant improvement in patellar tilt, with the median preop tilt of 9.5° ± 20.08 reducing to 3.2° ± 4.07, yielding a median improvement of 11.3° ± 21.26. These findings underscore the efficacy of the combined approach in optimizing patellar tracking and alignment while effectively addressing instability.

### Outcomes

Implant survivorship was excellent, with no cases of recurrent instability, maltracking, dislocations, infections, arthrofibrosis, or the need for manipulation under anesthesia. All patients achieved full range of motion (0–129.5 ± 8.3°), including one patient who sustained a delayed, traumatic periprosthetic patellar fracture at 10 months postoperatively following a mechanical fall. Aside from this single traumatic periprosthetic fracture, no postoperative complications, readmissions, or revision surgeries were reported at a minimum of 24 months.

One patient (ID #6) sustained a periprosthetic patella fracture 10 months after surgery following a slip while exiting a pool. Intraoperatively, the patellar button was noted to be securely fixed, and the fracture appeared to have occurred through one of the peg holes in the patella, potentially aligning with a docking site from the previous MPFL reconstruction. The trochlear component was stable and well-fixed. Treatment involved open reduction and internal fixation (ORIF) of the patella, retinacular repair, and quadriceps advancement.

Approximately 4 months after this initial ORIF, the same patient (ID #6) sustained a second traumatic injury after slipping and falling while walking in the rain, resulting in a fracture of the superior pole of the patella with associated proximal quadriceps migration and extensor mechanism disruption. Intraoperative findings revealed a new fracture line in the superior pole of the patella, distinct from the previous fracture, along with complete disruption of the medial and lateral retinacula. The fractured superior pole fragment was small, and the components remained well-fixed. The extensor mechanism was repaired without augmentation. The patient did not require revision of his components during either surgery.

**Figure 2:**
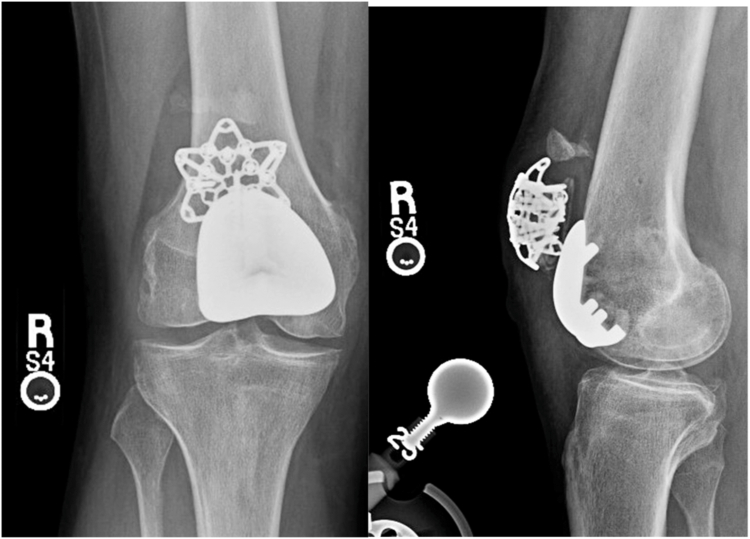
Postoperative anterior-posterior (AP) and lateral radiographs after patella open reduction internal fixation (ORIF) and extensor mechanism repair.

The patient is doing well 1 year after his most recent revision surgery, with no pain or functional limitations. He is ambulating independently, without assistance or a brace. On examination, he has full range of motion, no extensor lag, and central patellar tracking. His x-rays, as shown in Figure 2, demonstrate good alignment with no evidence of loosening.

### Patient-reported outcome measures

Preoperative and postoperative PROM scores are shown in Table 4. The mean preoperative Knee Injury and Osteoarthritis Outcome Score Joint Replacement score was 60.3 ± 13.7, improving by 14.7 ± 11.1 to a final score of 75.0 ± 12.3 (minimal clinically important difference [MCID] = 6.6). The PROMIS GPH score increased from 45.0 ± 6.8 to 48.3 ± 6.9, with a mean improvement of 3.3 ± 5.4 (MCID = 2.3). The PROMIS Global Mental Health score improved by 2.8 ± 4.2, from a baseline of 50.9 ± 10.4 to 53.7 ± 9.7. The Forgotten Joint Score at the last follow-up was 30.7 ± 25.8. The MCID was exceeded in all categories available.

**Table 4:** Preoperative and postoperative patient-reported outcome measures, including KOOS JR, PROMIS GPH, PROMIS GMH, and FJS.

PROM	Preop score mean (SD)	Mean difference from preop to last postop mean (SD)	Score at last F/U	MCID
KOOS JR	60.3 (13.7)	14.7 (11.1)		6.6 (Khalil TKA)
PROMIS GPH	45.0 (6.8)	3.3 (5.4)		2.3 (Khalil TKA)
PROMIS GMH	50.9 (10.4)	2.8 (4.2)		
FJS			30.7 (25.8)	

KOOS JR, Knee Injury and Osteoarthritis Outcome Score Joint Replacement; GMH, Global Mental Health; FJS, Forgotten Joint Score; PROMIS, Patient Reported Outcomes Measurement Information System; GPH, Global Physical Health.

## Discussion

Our study is the first to present a series of combined PFA and patella stabilization surgeries performed by 2 fellowship-trained surgeons. This approach addresses a historically challenging patient population with concomitant patellofemoral arthritis and patella instability. Traditionally, these patients experience chronic pain and functional limitations, often facing the difficult decision of either prolonged conservative management of symptoms or opting for early total knee arthroplasty (TKA), which may lead to the need for revision at a younger age. Our findings demonstrate that this combined approach yields favorable early outcomes with a low overall complication rate, offering a promising alternative to traditional treatment options.

Historically, outcomes of PFA exhibited considerable variability, primarily due to suboptimal implant designs and surgical techniques [10,11]. However, advancements in second-generation implants have significantly improved these outcomes, establishing PFA as a viable intervention for patients with isolated patellofemoral arthritis [[12], [13], [14], [15], [16]]. The benefits of PFA include preservation of bone stock, reduced operative times, and accelerated recovery, making it a preferred option when feasible. This is especially relevant in our study, where most patients with coexisting patellar instability and patellofemoral arthritis are relatively young. Furthermore, if necessary, conversion from PFA to TKA yields positive outcomes and is technically similar to primary TKA [17].

A systematic review reported survivorship rates of 91.7%, 83.3%, 74.9%, and 66.6% at 5, 10, 15, and 20 years, respectively. Notably, studies published after 2010 had a decrease in the annual revision rate from 2.33% to 1.93% [5]. These findings suggest improved outcomes and survivorship with the integration of second-generation implant systems. Although survivorship is improving, common indications for PFA revision include the progression of osteoarthritis and patellar maltracking, with the latter accounting for over 10% of failures in a review of more than 900 failed PFAs [5]. This is precisely the failure mechanism that our technique aims to prevent.

A study involving 609 patients with at least one patellar dislocation demonstrated a higher incidence of patellofemoral arthritis. Projections indicate that, at a 25-year follow-up, nearly 50% of patients with patellar instability will develop this condition, compared to only 8% of those without a history of dislocation or subluxation [18]. In this context, patellar instability is a significant risk factor and a coexisting condition for isolated patellofemoral arthritis.

To address the issue of maltracking, second-generation implants incorporate anterior femoral cuts similar to those used in TKA. These modifications improve patellar tracking by expanding the trochlear surface and promote proper valgus alignment [19]. These implants were also designed to be used in conjunction with MPFL reconstruction or tibial tuberosity medialization [4].

A study by Brusalis et al. involving 13 patients with both patellofemoral arthritis and lateral patellar instability found that combined MPFL reconstruction and PFA with short-term follow-up significantly improved patellar tilt. Specifically, the study found a mean improvement of 16 degrees in patellar tilt, along with favorable changes in various PROMs [7]. Our study corroborates these findings, demonstrating that a combined approach improved patellar tilt by a median of 11.3° degrees. Additionally, patient-reported outcomes improved across all participants.

In our series, a single complication was observed: one patient sustained 2 periprosthetic fractures following 2 separate falls. Although this event was likely beyond our control, it underscores a potential risk associated with the combined procedure, particularly due to the thinning of the patella and the proximity of the suture anchor to the arthroplasty peg. The patient underwent ORIF and extensor mechanism repair and has since experienced no further complications. Notably, the patient did not require component revision, augmentation of the extensor mechanism, or conversion to TKA. No other complications, revision surgeries, or manipulations under anesthesia were required, and no recurrent instability was observed. All patients successfully regained full range of motion.

The limitations of our study are those inherent to its retrospective design, including the absence of randomization. Additionally, the small sample size, short-term follow-up, and inclusion of 2 different types of patella realignment procedures may reduce the generalizability of our conclusions. The lack of functional strength testing further limits our ability to comprehensively assess functional outcomes. Importantly, we did not include a comparison group of patients undergoing isolated PFA, which limits our ability to determine whether the addition of a stabilization procedure influenced complication rates, patient-reported outcomes, or implant survivorship. Despite these limitations, the strengths of our study include the use of a uniform PFA implant across all patients, the consistent application of the same surgical technique for each case, and the involvement of the same, 2, fellowship-trained surgeons at a single institution. This consistency in surgical technique and surgeon expertise minimizes potential sources of variability and strengthens the reliability of our outcomes.

## Conclusions

Our study contributes promising evidence to the growing body of literature supporting the combined approach of PFA and patella stabilization. A unique aspect of our study is the collaboration of 2 specialty-trained surgeons, each performing their respective procedures. This combined technique may enhance both patient outcomes and implant survivorship, potentially delaying or even obviating the need for TKA. Additional advantages include reduced anesthesia exposure, a quicker return to normal activities, and a more accelerated rehabilitation protocol, particularly for range of motion, due to the minimized risk of patellar dislocation. While these results are encouraging, further large-scale studies with longer-term follow-up are necessary to fully assess the long-term efficacy and safety of this technique.

## CRediT authorship contribution statement

**Paul B. Walker:** Writing – review & editing, Writing – original draft, Methodology, Investigation, Formal analysis. **Lisa Su:** Writing – review & editing, Writing – original draft, Methodology, Investigation, Formal analysis, Data curation, Conceptualization. **Mathangi Sridharan:** Writing – review & editing, Resources, Formal analysis. **Murray Wong:** Supervision, Resources, Methodology, Investigation, Conceptualization. **Matthew Dipane:** Formal analysis, Data curation. **Guillermo Araujo-Espinoza:** Resources, Investigation, Formal analysis, Data curation. **Kristofer J. Jones:** Writing – review & editing, Supervision, Methodology, Investigation, Formal analysis, Conceptualization. **Adam A. Sassoon:** Writing – review & editing, Supervision, Project administration, Methodology, Investigation, Formal analysis, Conceptualization.

## Conflicts of interest

A.A. Sassoon is a paid consultant for Smith & Nephew, Zimmer Biomet, Biocomposites, and OrthAlign; receives research support from Biocomposites; holds stock in OrthAlign and Overture; and is a board or committee member for AAHKS. K.J. Jones is a paid consultant for Arthrex, Inc., JRF Ortho, and Vericel Corporation; receives research support from Aesculap/B. Braun, the Musculoskeletal Transplant Foundation, and Organogenesis; serves as a board or committee member for the American Orthopaedic Society for Sports Medicine, *The Journal of Bone and Joint Surgery (Am)*, and the NFL Musculoskeletal Injury Committee; and holds stock or stock options in Sparta Biopharma; all other authors declare no potential conflicts of interest.

For full disclosure statements refer to https://doi.org/10.1016/j.artd.2025.101951.

## Appendix A Supplementary data

Conflict of Interest Statement for Sassoon

Conflict of Interest Statement for Araujo-Espinoza

Conflict of Interest Statement for Jones

Conflict of Interest Statement for Su

Conflict of Interest Statement for Dipane

Conflict of Interest Statement for Sridharan

Conflict of Interest Statement for Wong

Conflict of Interest Statement for Walker
